# A modified persian version of the self-stigma of depression scale among the Iranian population: a methodological study in 2023

**DOI:** 10.1186/s40359-024-01802-w

**Published:** 2024-05-26

**Authors:** Alireza Jafari, Mahdi Moshki, Fatemehzahra Naddafi, Elaheh Lael-Monfared, Mahbobeh Nejatian

**Affiliations:** 1 Department of Health Education and Health Promotion, School of Health, Social Development and Health Promotion Research Center, Gonabad University of Medical Sciences, Gonabad, Iran; 2 Department of Health Education and Health Promotion, School of Health, Social Development and Health Promotion Research Center, Gonabad University of Medical Sciences, Gonabad, Iran; 3 Student Research Committee, Gonabad University of Medical Sciences, Gonabad, Iran; 4 Department of Health Education and Health Promotion, School of Health, Mashhad University of Medical Sciences, Mashhad, Iran; 5 Social Determinants of Health Research Center, Gonabad University of Medical Sciences, Gonabad, Iran

**Keywords:** Mental health, Psychometric, Stigma, Validity, Reliability, Depression

## Abstract

**Introduction:**

This cross -sectional research evaluated the psychometric properties of the Self-Stigma of Depression Scale (SSDS) among Iranian people.

**Methods:**

This methodological study was conducted among 881 people in 2023, Iran. The method of proportional stratified sampling was used to select participants. To evaluate the validity, face, content, construct, convergent, and discriminant were evaluated. The reliability of SSDS was assessed with the McDonald’s omega coefficient, Cronbach α coefficient, and test– retest (Intraclass Correlation Coefficient).

**Results:**

In confirmatory factor analysis, the factor loading of all items of SSDS was more than 0.5, and two items had low factor loading. After deleted these items, goodness of fit indexes (such as GFI = 0.945, RMSEA = 0.067, AGFI = 0.917, CFI = 0.941, RFI = 0.905) confirmed the final model with 14 items and four factors of social inadequacy (3 items), help-seeking inhibition (4 questions), self-blame (3 questions), and shame (4 questions). In the reliability phase, for all items of SSDS, Cronbach α coefficient was 0.850, the McDonald omega coefficient was 0.853, and the intraclass correlation coefficient was 0.903.

**Conclusion:**

The Persian form of SSDS was approved with 14 items and four factors: social inadequacy, help-seeking inhibition, self-blame, and shame. This tool can be used to check the status of self-stigmatization of depression in different groups.

## Introduction

Depression is a serious challenge in the field of public health and is considered the most common mental disorder [1, 2]. Depression refers to constant sadness and loss of interest in performing previous activities for long periods of time [3]. 4.4% of the world’s population and between 8% and 20% of Iranians suffer from depression. The prevalence of the disease in Iran has also increased and accounts for 35–45% of mental illnesses [4–6].

Genetic and epigenetic factors, various stresses, environmental factors, childhood abuse, and the absence of some nutrients cause depression [7]. Depression has negative effects on different physical, mental, and social dimensions of health. This disorder is associated with increased risk of cardiovascular disease, endocrine dysfunction, decreased social performance, reduction in quality of life, and increased death [8–12]. Overall, depression, as a serious and common disorder, not only affects all aspects of a person’s life and is a major burden on the family and society [13–15].

Depression is a treatable disorder, and drug therapy, psychotherapy, or a combination of both are evidence -based and effective treatments for depression. However, despite the availability of therapeutic approaches, there is a wide gap in this regard. In fact, a significant number of depression patients do not seek professional assistance. Statistics showed that in developing countries such as Iran, only 10–15% of mentally ill patients search for professional assistance; therefore, identifying barriers to help seeking is required [1, 16–19].

One of the barriers to help seeking in depression is self-stigma or personal stigma [20, 21]. Self-stigma refers to the innerness of shameful beliefs by a person with a mental illness that often causes deep shame and sin and may endanger the process of helping and treating a person [22, 23]. Self-stigma is common among people with depression, and approximately 20% of people have medium to high self-stigma [24]. Self-stigma leads to shame, despair, low self-esteem, reduced quality of life, decreased self-efficacy, inability, and suicide thoughts [25–27].

In the Barney study, depression stigma and its effect on Australian adults were investigated. The results showed that in most people, there is a sense of embarrassment and shame for requesting help, and this can lead to the reluctance of people to ask for help [21]. In addition, the results of the study in Iranian people showed a high prevalence of self-stigma [28]. Despite the importance of self-stigma in depression, there is a weakness in the study of its prevalence and nature [29]. The lack of a specific and valid tool is one of the possible reasons. Therefore, to determine the prevalence and severity of depression self-stigma in society, planning and implementing interventions to reduce depression self-stigma, it seems necessary to have a valid and reliable tool [30].

Data collection is an important step in any research and requires the use of appropriate tools [31]. The availability of a valid and reliable tool is essential for the study and helps the research team collect accurate and more credible information. Careful collection of information in any target group can help better design and execute preventive programs [32, 33]. Based on the review of the available literature, there was no consistent questionnaire about self-stigma of depression in the Iranian population, and one of the best tools to investigate depression is the Self-Stigma of Depression Scale (SSDS) [29]. The SSDS was designed and psychometrically evaluated in 2010 by Barney et al. [29]. The SSDS consists of 16 questions that survey the status of social inadequacy, help-seeking inhibition, self-blame, and shame. The validity and reliability of this questionnaire has been measured in both healthy and depressed populations and has been translated into several languages [29, 34, 35].

Given the negative effects of self-stigma on people’s tendency to receive psychological aid and the importance of obtaining these aids in the early stages of the disease [36, 37], the presence of a specific and appropriate tool in the community for measuring self-stigma levels is especially necessary. In the Iranian community, there was no suitable tool capable of dedicated depression self-stigma. Therefore, this study was conducted to determine the psychometric properties of the self-stigma of depression in the Iranian public population.

## Methods

This methodological study was conducted to evaluate the psychometric properties of the SSDS among Iranian general population in Gonabad city (Iran). Data were collected from February 2023 to July 2023.

### Sample size

To conduct factor analysis, a sample size of 500 is good [38, 39]. Therefore, in this study, confirmatory factor analysis (CFA) was assessed in 881 people.

### Sampling

The method of proportional stratified sampling was used to select participants. Each health center (*n* = 3) was considered as a stratum, and the required sample of each center was determined by the population of each center. People usually have a health record in health centers. Initially, people with the entry criteria were identified through the list of each center, and then the required samples were selected using a simple random method. The questionnaires were completed in the health centers, and if the person failed to visit the health centers, the necessary coordination was made and the questionnaire was administered in person. People who were 18 years and older, lived in Gonabad for more than a year, and tend to participate in the study were selected and entered into the study.

### Instruments

#### Self-stigma of depression scale (SSDS)

This tool was designed by Barney et al. [29] and has 16 items and 4 subscales of social inadequacy (4 questions), help-seeking inhibition (4 questions), self-blame (4 questions), and shame (4 questions). It is a good scale to survey the status of self-stigma of depression. All questions of SSDS are measured using the five -choice Likert scale (“completely disagree” to “completely agree”). In Barney study, Cronbach alpha coefficient of SSDS and subscales of social inadequacy, help-seeking inhibition, self-blame, and shame were 0.87, 0.79, 0.79, 0.78, 0.83, respectively [29].

### Translation, cultural adoption, and validity section

Before starting the translation of the SSDS, in the first step, the required permission was obtained from the SSDS designer. The processing of translation (forward and backward) of SSDS was performed based on the WHO Guidelines [40]. In the forward translation, the English version of SSDS was translated to the Persian version by 2 translators. These translated Persian versions of SSDS were compared together and then one version was created. In backward translation, the Persian version of SSDS was translated to the original language by two translators and compared with the original English version.

### Validity

The validity of the final translated Persian version of SSDS was evaluated with face validity, content validity, construct validity, convergent validity, and discriminant validity.

#### Face validity

In this step, the qualitative face validity of the final translated Persian version of SSDS was evaluated by 9 experts (specialists of Psychology and specialists of Health Education and Health Promotion). The questionnaire was also given to 10 members of the target group and reviewed.

#### Content validity

Qualitative and quantitative methods were used to survey content validity. The Persian version of the SSDS was evaluated by 9 experts (specialists of Psychology and specialists of Health Education and Health Promotion) and two methods of content validity ratio (CVR) and content validity index(CVI) were used to check the quantitative content validity [41].

##### CVR

In this section, each item was assessed with a three-point scale of “essential”, “useful but it is not necessary” and “it is not necessary” and below formula (N = number of experts, nE = Number of experts that selected “essential”) was used to calculate the CVR. Given that the number of evaluators was 9, the CVR is acceptable if the score is above 0.75 [42].

$$CVR=\frac{{n}_{E}-\left(N/2\right) }{N/2}$$

##### CVI

Each item of the SSDS was assessed using a four-point Likert scale in terms of relevance or specificity, clarity or transparency, and simplicity and fluency based on the following formula: A score of 0.78 or more is acceptable [43].

$$CVI=\frac{Number of \text{e}\text{x}\text{p}\text{e}\text{r}\text{t}\text{s} selected items of 3 and 4}{Total number of \text{e}\text{x}\text{p}\text{e}\text{r}\text{t}\text{s} }$$

#### Construct validity

In this study, because SSDS was adopted as a standard scale and the aim of this study was not to identify a new conceptual structure, only CFA was performed. In the CFA part, all factors were evaluated by AMOS software version 24. In CFA, goodness-of-fit indexes need to be reviewed to confirm the final model. The final model will be confirmed if the goodness-of-fit indexes are standard. In this study, the following goodness of fit indexes were used: parsimonious normed fit index (PNFI > 0.5), relative fit index (RFI > 0.9), chi-square ratio to degree of freedom (χ2/df < 5), comparative fit index (CFI > 0.9), adjusted goodness of fit index (AGFI > 0.9), normed fit index (NFI > 0.9), parsimony comparative fit index (PCFI > 0.5), parsimony goodness of fit index (PGFI > 0.5), root mean square error of approximation (RMSEA < 0.08), tucker Lewis index (TLI > 0.9), incremental fit index (IFI > 0.9), and goodness of fit index (GFI > 0.9) [44–48].

#### Convergent and discriminant validity

To survey the convergent validity of SSDS, the average variance extracted (AVE) was calculated. To assess the discriminant validity of SSDS, MSV (maximum shared squared variance) and ASV (average shared squared variance) were calculated. AVE is more than 0.5 show acceptable convergent validity, and MSV and ASV AVE show acceptable discriminant validity [48].

### Reliability

Test-retest reliability of SSDS was calculated by intraclass correlation coefficient (ICC) among 47 participants twice with a period of one month. To check the internal consistency, McDonald’s omega coefficient was assessed by software JASP version 0.11.1.0 and the Cronbach α coefficient was assessed by software SPSS version 23.

### Statistical analysis

Cronbach alpha coefficient and ICC were calculated using SPSS version 23 software. The software JASP Version 0.11.1.0 was used to calculate McDonald’s omega coefficient. In addition, the CFA was assessed by AMOS software version 24. In addition, the standard error of measurement (SEM = SD×√(1-ICC)) and smallest detectable change (SDC = SEM×1.96×√2) [49–51] were assessed for SSDS and subscales of social inadequacy, help-seeking inhibition, self-blame, and shame.

## Results

### Demographic characteristics

The average (SD) age of the participants was 33.60 (13.22). Among the participants, 51.2% (*n* = 451) were female and 48.8 (*n* = 430) were male. In terms of marital status, 55.3% (*n* = 487) were married and 44.7% (*n* = 394) were single. Among participants, 35.6% (*n* = 314) had a bachelor’s degree. Only 16.3% (*n* = 143) of participants had a history of mental disorder, and only 18% of participants (*n* = 159) were referred to a psychologist (Table 1).

**Table 1:** Frequency distribution of demographic characteristics (*n* = 881)

Variables		*N*	%
**Sex**	Male	430	48.8
	Female	451	51.2
**Occupation**	Housewife	104	11.8
	University student	345	39.2
	Employed	219	24.9
	Retired	48	5.4
	Self-employed	126	14.3
	laborer	21	2.4
	Unemployed	18	2
**Marital status**	Married	487	55.3
	Single	394	44.7
**Education level**	Illiterate	2	0.2
	Elementary school	19	2.2
	Middle school	25	2.8
	High school	37	4.2
	Diploma	255	28.9
	Associate degree	125	14.2
	Bachelor’s degree	314	35.6
	Master’s degree	81	9.2
	PhD	23	2.6
**History of mental disorder**	Yes	143	16.3
	No	737	83.8
**Refer to psychologist**	Yes	159	18
	No	722	82
**Refer of your family to psychologist**	Yes	162	18.4
	No	571	64.8
**Mental information**	Yes	659	74.8
	No	222	25.2

### Face and content validity

In the qualitative face validity, 3 items were modified, and in the qualitative content validity, 2 items were modified. The rates of CVR and CVI for all items of SSDS were 0.773 and 0.923, respectively.

### CFA section

**Fig. 1:**
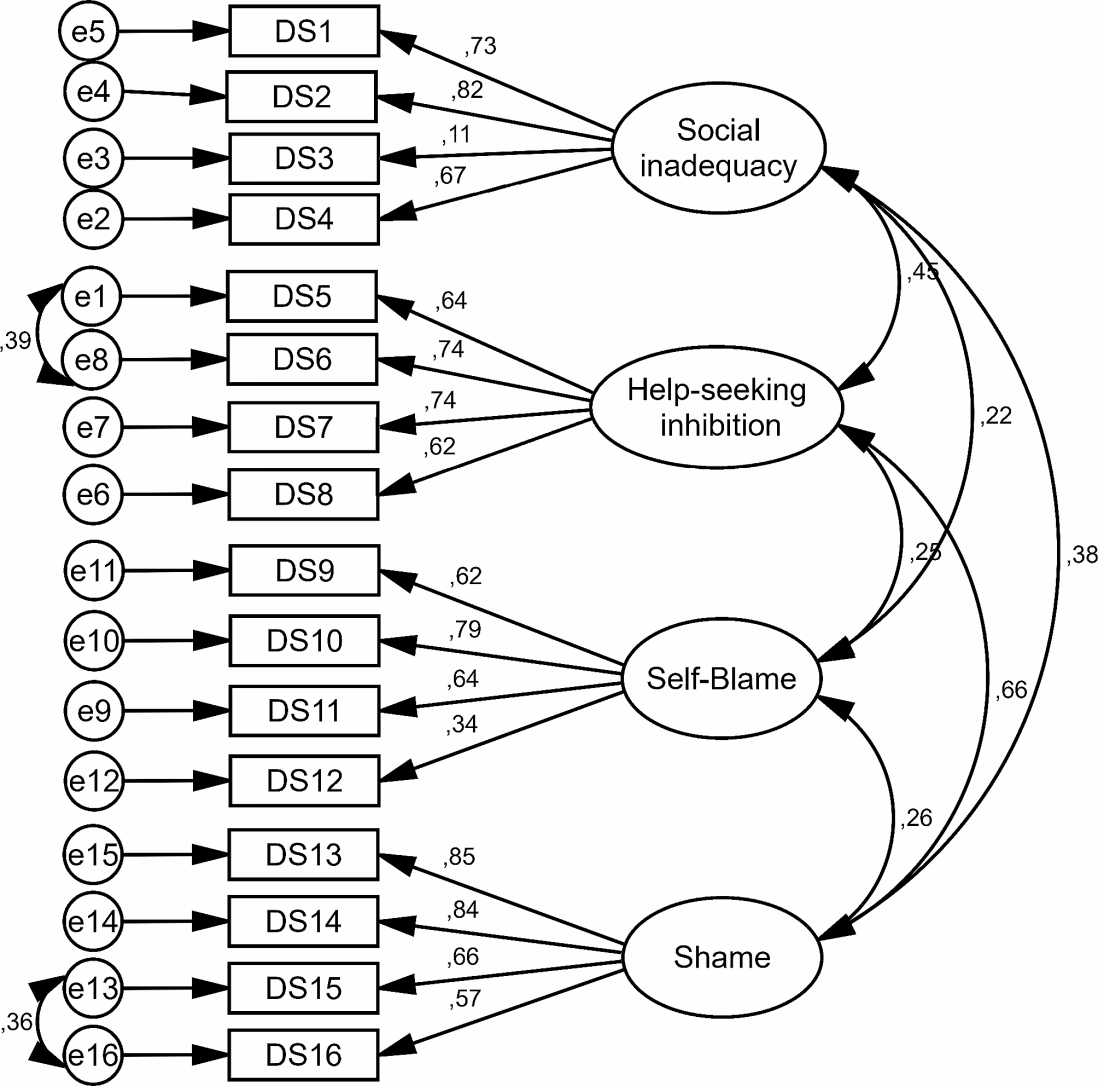
Standardized parameter estimates for the factor structure of the Persian version of SSDS (The first model with 16 items)

**Fig. 2:**
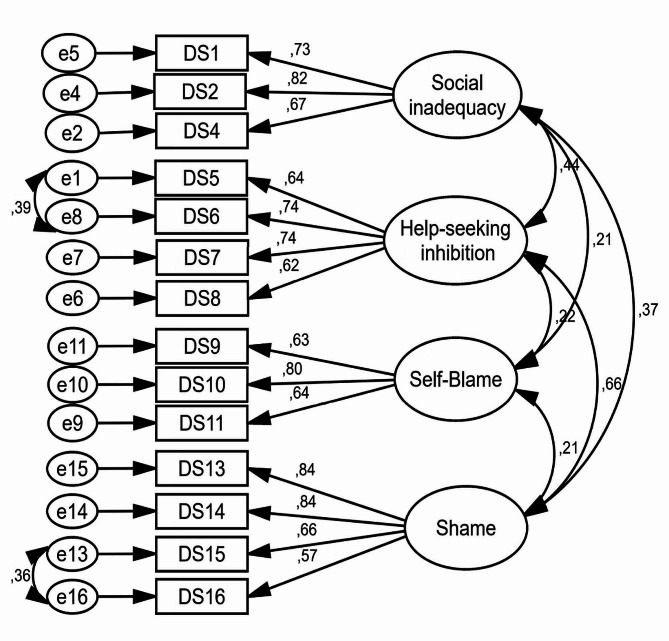
Standardized parameter estimates for the factor structure of the Persian version of SSDS (The second model with 14 items and deleted of 2 items)

In the first model, all items of SSDS were survey, and factor loading of two items (item 3 and item 12) was low (less than 0.4), and the goodness of fit indexes of the model were not appropriate (Table 2; Fig. 1). In the second model, after the deleted of the two items with low factor loading (item 3 and item 12), the goodness of fit indexes of the model were improved (such as RMSEA = 0.067, AGFI = 0.917, NFI = 0.928) and showed that the final model of SSDS had an acceptable value (Table 2; Fig. 2). The SSDS with 14 items and four factors of social inadequacy (3 questions), help-seeking inhibition (4 questions), self-blame (3 questions), and shame (4 questions) (Table 3) was confirmed (Table 3). The minimum score of SSDS is 14 and maximum is 70, and a high score indicate a higher self-stigma of depression.

**Table 2:** The model fit indicators of the Persian version of lo SSDS

Goodness of fit indices	Confirmatory factor analysis of first model	Confirmatory factor analysis of second model (Final model)	Acceptable value
χ2	650.566	344.394	-
df	97	69	-
X^2^/df	6.707	4.991	< 5
*P*-value	0.000	0.000	*p* > 0.05
IFI	0.889	0.941	> 0.9
GFI	0.912	0.945	> 0.9
RMSEA	0.081	0.067	< 0.08
CFI	0.889	0.941	> 0.9
PNFI	0.705	0.703	> 0.5
AGFI	0.876	0.917	> 0.9
PGFI	0.650	0.621	> 0.5
PCFI	0.718	0.714	> 0.5
NFI	0.872	0.928	> 0.9
RFI	0.842	0.905	> 0.9
TLI	0.862	0.922	> 0.9

**Table 3:** Factor loadings of the Persian version of SSDS

Subscales	Items Participants were asked to indicate how they would think of or feel about themselves if they were depressed…	Factor loadings
Social inadequacy	DS1: I would feel I couldn’t contribute much socially.	0.733
	DS2: I would feel inadequate around other people.	0.816
	*DS3: I would feel like I was good company.*	*Deleted*
	DS4: I would feel like a burden to other people.	*0.671*
Help-seeking inhibition	DS5: I would think I should be able to cope with things.	0.644
	DS6: I would think I should be able to ‘pull myself together’.	0.744
	DS7: I would think I should be stronger.	0.740
	DS8: I would think I only had myself to blame.	*0.622*
Self-blam	DS9: I would think I should be able to cope with things.	0.625
	DS10: I would think I should be able to ‘pull myself together’.	0.801
	DS11: I would think I should be stronger.	0.640
	*DS12: I would think I only had myself to blame.*	*Deleted*
Shame	DS13: I would feel ashamed.	0.845
	DS14: I would feel embarrassed	0.836
	DS15: I would feel inferior to other people	0.662
	DS16: I would feel disappointed in myself	0.573

### Convergent and discriminant validity

In Table 4, the results of discriminant validity and convergent validity were mentioned. Only two subscales of social inadequacy and shame had the acceptable AVE. Also, the MSV and ASV had the rate less than AVE (Table 4).

**Table 4:** Results of convergent and discriminant validity of the Persian version of SSDS

Subscales	CR	AVE	MSV	ASV
Social inadequacy	0.785	0.551	0.193	0.083
Help-seeking inhibition	0.783	0.476	0.438	0.225
Self-Blame	0.733	0.481	0.047	0.045
Shame	0.824	0.545	0.438	0.205

### Reliability section

For total SSDS, Cronbach α coefficient was 0.850, McDonald’s omega coefficient was 0.853, and ICC was 0.903. The results of scale reliability for each factor of SSDS were mentioned in Table 5. The standard error of measurement and the smallest detectable change in SSDS were 2.581 and 7.154, respectively (Table 5).

**Table 5:** Descriptive statistics of the Persian version of SSDS

Subscales	Item	Range	Cronbach α coefficient	McDonald’s omega coefficient	Intraclass Correlation Coefficient (ICC)	95% Confidence Interval		*P*-value	SEM	SDS
						Lower Bound	Upper Bound			
Social inadequacy	3	3–15	0.874	0.882	0.890	0.801	0.939	< 0.001	0.945	2.619
Help-seeking inhibition	4	4–20	0.813	0.824	0.932	0.879	0.962	< 0.001	0.805	2.231
Self-Blame	3	3–15	0.784	0.811	0.860	0.747	0.922	< 0.001	0.883	2.447
Shame	4	4–20	0.861	0.865	0.828	0.690	0.904	< 0.001	1.505	4.171
Total SSDS	14	14–70	0.850	0.853	0.903	0.825	0.946	< 0.001	2.581	7.154

### Ceiling and floor effects

Results of this study did not show ceiling and floor effects because less than 15% of respondents got lowest and highest scores. 0.7% (*n* = 6) of the participants got the lowest score and 1% (*n* = 9) got the highest score.

## Discussion

The self-stigma of depression is one of the major obstacles to help seeking and treatment. However, a few studies have been examined in Iran, and one of the possible causes could be the lack of a specific tool for measuring the self-stigma of depression. As a result, the present study was conducted in Iran’s public population aimed at translating, cultural matching, and evaluating the validity and reliability of SSDS. The results showed that the modified Persian version of the SSDS has good construct validity and reliability. In relation to construct validity, the two items were deleted in CFA due low factor loading, and the modified Persian version of SSDS with 14 items and 4 four factors was confirmed with appropriate goodness of fit indices. In terms of reliability, the results showed that the modified Persian version of SSDS had appropriate internal and external reliability, and Cronbach alpha coefficient, McDonald’s omega coefficient, and ICC were 0.850, 0.853, and 0.903, respectively.

Psychometric properties of SSDS were evaluated in the Germans and in CFA and based on the goodness of fit indexes (χ2/df = 2.133, TLI = 0.989, CFI = 0.991, SRMR = 0.072, RMSEA = 0.080), the SSDS was confirmed in the German people with 16 items and 4 factors [52]. A study among Australians evaluated the validity and reliability of the SSDS with 16 items and 4 factors in three target groups: university students, people with depression, and the public population. The reliability of SSDS was appropriate and the results of construct validity were similar in all three groups, which may indicate that self-stigma of depression is not associated with the experience of depression, but is related to community beliefs about how to deal with depression [29].

A study assessed the validity and reliability of the Turkish version of the SSDS in a population of depressed and non -depressed people. In this study, the item “I would feel like I was good company” was removed. In CFA, based on the goodness of fit indexes (CFI = 0.890, RMSEA = 0.080), SSDS was confirmed with 15 items and 4 factors. Eventually, the Turkish version also had good validity and reliability (Cronbach α coefficient = 0.847) [53]. In our study, two items of “I would feel like I was good company”, and “I would think I only had myself to blame” were removed due to low factor loadings. In the psychometric evaluation of the Turkish version of the SSDS, these two items did not have the appropriate indicators, and finally, the item “I would feel like I was in good company” was removed from the Turkish version. Due to the translation of the tools by the two separate teams and in two different languages, translation problems or misunderstandings cannot be described as the reason for the inappropriate indicators of these two items. Also, the psychometric properties of the two Turkish and Persian versions were performed in the two relatively different target populations in terms of cultural and depression experience, and culture or the type of target population will probably not be the reasons for this issue.

In our study, the first factor was social inadequacy, which was confirmed with three items, and one item (I would feel like I was good company) was removed due to low factor loading. The Cronbach alpha coefficient, McDonald’s omega coefficient, and ICC for this factor were 0.874, 0.882, and 0.890, respectively. Cronbach alpha coefficient of this factor in the Australian and Turkish versions were 0.79 and 0.822, respectively [29, 53]. Severe disorders in social functioning are complications of depression disorders [54]. In a qualitative study by Prizeman et al., youths with depression described self-stigma as a factor that disrupts social relationships and provokes symptoms of depression, isolation and loneliness [55]. As a result, SSDS can be helpful in measuring social inadequacy.

The second factor was help-seeking inhibition, which was confirmed with 4 items. The Cronbach α coefficient, McDonald’s omega coefficient, and ICC for this factor were 0.813, 0.824, and 0.932, respectively. Cronbach alpha coefficient were 0.79 and 0.686 in the Australian and Turkish versions [29, 53]. According to Schomerus et al., self-stigma of depression is not only an important obstacle in help seeking, but also reduces perceived need for assistance and disrupts the assessment of depression symptoms [20].

The third factor was self-blame, which was confirmed with three items, and one item (I would think I only had myself to blame) was removed due to low factor loading. The Cronbach α coefficient, McDonald’s omega coefficient, and ICC for this factor were 0.784, 0.811, and 0.860, respectively. Cronbach α coefficient were 0.78 and 0.637 in the Australian and Turkish versions, respectively [29, 53]. Finally, the fourth factor was shame, which was confirmed with 4 items. The Cronbach α coefficient, McDonald’s omega coefficient, and ICC for this factor were 0.861, 0.865, and 0.828, respectively. The Cronbach alpha coefficient of this factor in the Australian and Turkish versions were 0.83 and 0.854, respectively [29, 53]. Self-stigma is a product of emotional internalization such as blame and shame [56]. Excessive blame is one of the main symptoms of major depressive disorder [57]. Also, the feeling of shame can also follow self - blame [58]. A study showed that more than 80% of people with major depression disorder suffered from self - blame and subsequent shame. Therefore, accurate evaluation of self-blame is essential for classifying and diagnosing major depression disorder [59]. Given that SSDS measures self - blame and shame, its use can be useful in this regard.

In our opinion, the first three items of the third subscale (self-blame) have some main problems. First, these three items (i.e., I would think I should be able to cope with things, I would think I should be able to ‘pull myself together’ and I would think I should be stronger) are completely equivocal: One perception of these items is that I should accept my illness, make myself stronger and not feel weak and defeated because this illness is similar to other diseases, and it will be resolved by seeking help from specialists and undergoing treatment, and I will recover. This perception is not only self-blame but also the opposite of self-stigma. In this regard, it is recommended that necessary adjustments be made to these three items in future studies. For example, these items should have been written in this way: if I am depressed, I should make myself stronger and cope with things without needing treatment etc.

The second perception is that if others say that I have depression, I should be strong, deal with issues, and pull myself together because I’m not really depressed and I don’t have depression at all. I don’t need treatment. In fact, these labels and stigmas are not mine. This perception is completely in line with self-stigma, but the problem with this perception is that it is not self-blame, but rather extreme and harmful perfectionism. Therefore, it is suggested to modify the name of this subscale. It seems that the reason for removing the last item (I think I only blamed myself) from this subscale in the CFA stage, despite the fact that this item is both self-blame and self-stigma, is the above problems.

### Strengths and limitations

A limitation of this study was that depression experience and depressive symptoms in the target population were not measured. The strengths of this study were the large sample size and the measurement of various reliability indicators, including ICC and Omega McDonald’s, which were less calculated in previous studies. The study also checked the validity and reliability of SSDS in the general population and can therefore be used in various target groups, although psychometric evaluation of this scale is recommended in the population of people with a diagnosis of depression.

## Conclusion

The Persian form of SSDS was approved with 14 items and four factors: social inadequacy, help-seeking inhibition, self-blame, and shame. This tool can be used to check the status of self-stigmatization of depression in different groups. Surveying the status of the self-stigmatization of depression can be helpful in designing appropriate training programs.

## Data Availability

All data generated or analyzed during this study are included in this published article.

## Abbreviations

SSDS: Self-stigma of depression scale
CFA: Confirmatory factor analysis
CFI: Comparative fit index
PCFI: Parsimony comparative fit index
x2/df: Chi-square ratio to degree of freedom
AGFI: Adjusted goodness of fit index
GFI: Goodness of fit index
IFI: Incremental fit index
RMSEA: Root mean square error of approximation
NFI: Normed fit index
PNFI: Parsimonious normed fit index
PGFI: Parsimony goodness-of-fit index
TLI: Tucker Lewis index
RFI: Relative fit index
AVE: Average Variance Extracted
ASV: Average Shared Squared Variance
MSV: Maximum Shared Squared Variance
SEM: Standard Error of Measurement
SDC: Smallest Detectable Change
ICC: Intraclass Correlation Coefficient
